# Detection of Selected Heavy Metal Ions Using Organic Chromofluorescent Chemosensors

**DOI:** 10.3390/molecules30071450

**Published:** 2025-03-25

**Authors:** Samina Aslam, Iram Kousar, Sadia Rani, Wajiha Altaf, Sadia Bristy, Rachid Skouta

**Affiliations:** 1 Department of Chemistry, The Women University Multan, Multan 60000, Pakistan; kousariram361@gmail.com (I.K.); sadiarani112wb@gmail.com (S.R.); wajihaaltaf0@gmail.com (W.A.); 2 Department of Chemistry, University of Massachusetts, Amherst, MA 01003, USA; sbristy@umass.edu; 3 Department of Biology, University of Massachusetts, Amherst, MA 01003, USA

**Keywords:** heavy and transition metal (HTM) ions, chemosensor design, fluorescent chemosensors, colorimetric chemosensors, environmental monitoring, biological detection

## Abstract

Heavy and transition metal (HTM) ions have significant harmful effects on the physical environment and play crucial roles in biological systems; hence, it is crucial to accurately identify and quantify any trace pollution. Molecular sensors which are based on organic molecules employed as optical probes play a crucial role in sensing and detecting toxic metal ions in water, food, air, and biological environments. When appropriate combinations of conduction and selective recognition are combined, fluorescent and colorimetric chemosensors are appealing instruments that enable the selective, sensitive, affordable, portable, and real-time investigation of the possible presence of heavy and transition metal ions. This feature article aims to provide readers with a more thorough understanding of the different methods of synthesis and how they work. As noted in the literature, we will highlight colorimetric and fluorometric sensors based on their receptors into multiple categories for heavy metal ion detection, such as Hg^2+^, Ag^2+^, Cd^2+,^ Pb^2+^, and In^3+^, and simultaneous multiple-ion detection.

## 1. Introduction

**Figure 1:**
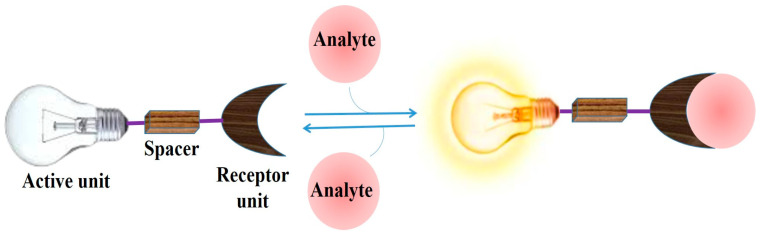
Diagrammatic illustration of an analyte’s chemosensor binding. Reproduced from ref. [12]. Analyte is added in the forward reaction, due to which the fluorescence is “on”. The analyte is detached from the analyte-receptor complex in the backward direction indicating that the fluorescence is “off”.

Heavy and transition metal (HTM) ions are critical to biological systems but pose significant threats to the physical environment as pollutants. Therefore, precise detection and quantification of trace-level contamination are essential for safe environmental and biological health [1,2]. The molecular recognition sensing of metal ions is one of the most vibrant and fascinating fields of research in analytical chemistry due to its major impact on industrial sector processes [3], environmental science [4], catalysis [5], medicine, biological and human sciences [6]. Molecular sensors, which are optical probes based on organic molecules, are essential for identifying and detecting detrimental metal ions in biological, food, water, and air environments [7]. A molecule must have a chromophore unit and appropriate active binding sites in order to function as a robust and efficient optical sensor [8]. The development in innovations of novel efficient chromofluorescent chemosensors for detecting toxic metal ions with high selectivity and sensitivity is currently an important field of research for many scientists [9]. The term “chemosensor” refers to “devices that bind selectively and reversibly to the active site of analyte with concomitant change in one or more properties of the system, such as fluorescence, color, or redox potential” [10,11]. As Figure 1 shows, a chemosensor typically consists of three parts: (1) a “receptor” or binding unit, (2) a “signaling unit” that alters its photophysical properties upon interacting with the analyte, and (3) a “spacer” that moifies the electronic interaction between the receptor and signaling unit.

Chemosensor agents have been applied to detect, quantify, and visualize various species including positively charged cations, negatively charged anions, or neutral molecules in real-world or simulated sample analysis. These chemosensor agents have been well evaluated and effective in identifying and quantifying the presence of special molecules in diverse sample analyses. A molecule must have a chromophore unit and suitable active binding sites in order to be an efficient optical sensor [8]. The contemporary research in chemosensor design development is focused on implementing various methodologies to use the optimal binding moiety for detection and quantification of selective metal analytes. This includes the strategic development of chemosensors with customized binding sites to facilitate special recognition and binding of the metal ions of interest [13]. Research is being conducted on a collection of organic, inorganic, and hybrid materials to make over extremely sensitive and selective sensors [14]. Organic compounds with nitrogen, oxygen, and sulfur moieties are used as chemosensors to detect and quantify different heavy metal ions because of their multidentate existence, which allows them to coordinate with different metal ions and produce quantitative analytical signals. These compounds also provide coordination sites to act as ligands in case of coordination chemistry [15].

It is usually accepted that some heavy metal ions are essential to many biological processes, including growth and metabolism [16]. Hg^2+^, Ni^2+^, Cu^2+^, Cd^2+^, Zn^2+^, Ag^+^, Pd^2+^, Pb^2+^, Fe^2+^, Cr^3+^, Pt^2+^, and Co^2+^ are significant metal ions among the heavy metal ion series [17] because they are frequently employed in industrial paving stones to produce a range of items that are beneficial to human beings for performing specific processes [18]. Excessive exposure to heavy metals can have toxic effects on the environment and human health [19]. One of the most hazardous contaminants being studied is heavy metal ions, which are directly associated with a wide range of human illnesses [20]. As anions play a vital role in biological and environmental ecosystems, their detection is also crucial. A certain anion may be advantageous in some applications if present in a common, reasonable amount, but not in a significant quantity [21]. Meanwhile, the detection of neutral substances usually entails characteristic biomolecules like nucleotides and amino acids. Therefore, the evolution of advanced chemical methodologies for their identification is necessary for worldwide public health. Common examples of focused analytical techniques for the identification and measurement of heavy metal ions are atomic absorption and emission spectrometry [22], capillary electrophoresis [23], high-performance liquid chromatography (HPLC) [24], voltammetry, and inductively coupled plasma atomic emission spectrometry (ICP-AES) [23]. These analytical tools typically demand expensive and complex equipment [25], skilled and competent operators, and extremely intricate protocols [26]. This severely restricts the practical use of these detection approaches, especially in areas with limited resources [27]. Therefore, it is crucial to design highly sensitive, easy, widely applicable, and cost-effective chemical procedures for accurately detecting metal ions in several materials [28]. The easiest methods are colorimetric and fluorometric organic chemosensors due to their superior selectivity, high sensitivity, and innate simplicity [29,30,31]. They do not require expensive or extremely complex equipment for the quantitative and qualitative detection of metal ions and other contaminants. However, the aforementioned chemosensors have a few drawbacks, such as multiple-ion selectivity or the inability to detect at the trace level in aqueous conditions [32]. Many organic colorimetric and fluorescent chemosensors have been designed with distinct sensing mechanisms to precisely identify different metal ions [33].

A number of methods are being used to construct the required binding moiety in a chemosensor to identify the targeted and interested analyte [34]. Because of their excellent photophysical characteristics, extremely low detection limit, great selectivity, synthetic flexibility, reversibility, biocompatibility, and use across a broad pH range, organic heterocyclic compounds are useful in the development of chemosensors. The ability of these compounds to interact with different metal ions and generate identifiable analytical signals makes them extremely important. These compounds contain donor atoms such as N, O, and S atoms [35]. Scientists are working to develop chemosensors employing a variety of organic compounds due to their requirement for metal ion detection [36]. Colorimetric sensors possessing imine and azide groups have been readily synthesized and have a potent ability to attach to metal ions. When these molecules with aryl substitution are combined, a conjugated system with absorbance near or in the visible-light spectrum region can be developed [37,38]. The amide group, which also contains thioamide, is a typical binding group for metal ion chemosensors [39]. The thiourea group also has the perfect signaling unit and binding moiety [40]. The fluorescent chemosensing method is superior to the other techniques in a number of ways such as sensitivity, specificity, and real-time monitoring with quick reaction times. In recent years, researchers have become interested in fluorescent sensors because of their many advantages, which include nondestructive sample analysis, low equipment costs, great sensitivity, and exceptional selectivity [41]. Fluorescence chemosensors are especially useful for in situ imaging of physiologically significant ions and tiny compounds [42]. A great deal of effort has been taken in order to rationally develop a fluorescence chemosensor for ions and neutral analytes [43]. Numerous attempts have already been made to synthesize fluorescent sensors that detect metal ions using thiol deprotonation [44,45] cyclization [46], and ring-opening [47] in thiourea derivatives [48]. The development of fluorescent probes that can remove a particular metal ion selectively without harming other metal ions is therefore still of interest [49,50]. In reaction to subtle changes in the environment, fluorescent sensors usually monitor their signal as a change in emission wavelength, fluorescence lifetime, or emission strength [39,51]. However, since metal ions can be detected even in live systems, which have been shown to exhibit distinct emission properties upon metal binding, the creation of highly selective ratio metric and “turn-on” fluorescence probes is increasingly crucial [52,53]. A simple Schiff base can be used to selectively identify ions in a semi-aqueous medium by using thiocarbohydrazide as a fluorescent chemosensor [54,55]. The great sensitivity and selectivity of chemosensors with hydrazine–hydrazide moieties in detecting a range of cations and anions make them desirable options [56,57]. The functional group –CO–NHN=CH– is present in hydrazide–hydrazones, a new chemosensor subclass of Schiff base compounds [58]. Furthermore, Schiff bases’ have strong affinity for metal ions which are frequently used in recognition, making them essential to binding chemistry [59]. They are hence superior chemosensors. Schiff-base fluorescence chemosensors are gaining a lot of attention due to the low cost of raw materials, variety of designs, and simplicity of synthesis [60]. These substances have long piqued interest in the development of novel chemosensors due to their wide range of medicinal properties, which include antibacterial, antiviral, antifungal, anticancer, and anti-inflammatory properties [61,62]. Thio/carbohydrazide identifies and quantifies organic and inorganic substances [63]. It is produced by reacting carbon disulfide with hydrazine hydrate [64]. Carbohydrazide with pyrazole and naphthaldehyde moieties creates multifunctional sensing compounds for metal ions and anions [65,66,67]. Thio/carbohydrazide forms colored complexes with metal ions, aiding in identification [68,69]. It is used in contact-print processes to detect metal ions [70,71]. Introducing a thio/carbazide group to sensor molecules enhances binding with transition and heavy metals [72]. The chemosensor comprises a binding site with three active functional groups (–OH, –C=S–, and –C=N–) that collaborate for identification and detection of various metal ions [73]. In general, chelation-induced or coordination-induced charge transfer mechanisms are employed to detect particular metal ions in a complex formation system. These mechanisms operate on the framework of the “lock and key” theory, through which the sensor molecule (the “lock”) is designed to bind with an identifiable cation (the “key”) selectively, generating a unique charge transfer response that offers discrimination between various cations [71,74]. Thus, interpreting the results demands an exact understanding of the host–guest chemistry, which has caused a great deal of fascination in the development of chemical and biological sensors. The sensitivity interaction relationships between hosts and guests have become an area of research focus, triggering innovation and development in the research of metal ion sensing technologies [75]. The “host” is frequently involved in a big chemosensor molecule featuring a central hole or cavity, including an enzyme or synthetic organic cyclic molecule. A tiny synthetic anion or a mono-atomic cation may be represented as the “guest”. The Lewis basic donor atom-containing chemosensor molecular entity with “convergent” binding sites is commonly referred to as the host. Lewis acid metal cations are examples of the “divergent” binding sites that have been found in the guest [76]. This research represents a broad overview of organic compounds functioning in colorimetric and fluorescent chemosensors for identification and detection of heavy metal ions, anions, and other environmentally hazardous substances. The main focus is on synthesis of Aryl-substituted organic-based chemosensors, which have shown great promise in detecting a wide range of metal ions including silver, cadmium, mercury, lead, magnesium, indium, and simultaneous multi-ion detection through colorimetric and fluorometric turn-off and turn-on response.

## 2. Detection of Heavy Metal Ions

Here, we have overviewed the monitoring and detection of several heavy metal ions by employing various types of organic chemosensors.

### 2.1. Hg^2+^ Ion Detection

**Scheme 1:**
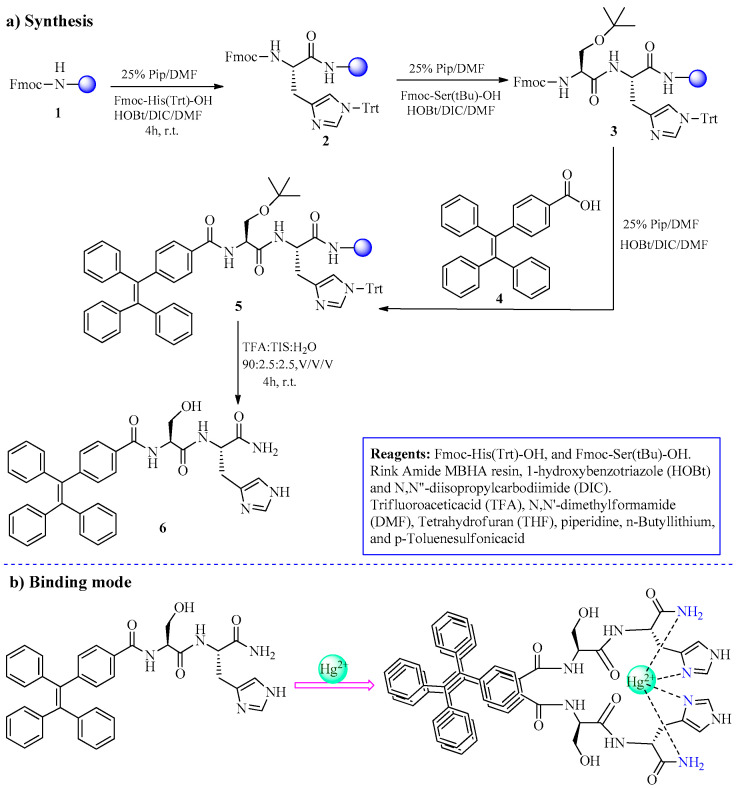
(**a**) Synthesis of solid-phase peptide-based sensor **6**; (**b**) Proposed binding mode.

Of all the HTM ions, mercury ions are among the most dangerous for the environment and human health. As a result, numerous kinds of fluorescent chemosensors for Hg^2+^ ions detection have been synthesized as fluorescence offers an accurate and effective analysis method [1,2,77,78]. Moreover, the need for a turn-on response to Hg^2+^ was great since a turn-off response would have been confused with a false signal caused by Hg^2+^ quenching, chemosensor precipitation, or impurity absorbance. Thus, there is a need to create fluorescent chemosensors that will trigger a reaction in response to detecting Hg^2+^ ions in aqueous solutions in a selective and sensitive manner [79,80,81,82,83,84,85,86,87,88,89,90,91,92]. A chemosensor based on the metal ions’ peptide receptor was developed by using an aggregation-induced emission fluorospore. It enables the detection of heavy metal ions in cell analysis and aqueous solution. The peptidyl chemosensor with tetraphenylethylene fluorophore demonstrated a completely selective turn-on response to Hg^2+^ among 16 metal ions in an aqueous buffered solution including NaCl. When Hg^2+^ ions were linked by the peptidyl chemosensor and subsequently collected in an aqueous buffered solution, the emissions at around 470 nm revealed a noticeable rise (OFF-ON). The fluorescent sensor responded to Hg^2+^ with excellent sensitivity since about 1.0 equivalent of Hg^2+^ was required for the shift in emission intensity to be saturated. Hg^2+^ ion detection limit of **5** was smaller than the EPA’s highest amount of Hg^2+^ permissible in drinking water. Furthermore, the peptidyl chemosensor entered living cells and used the turn-on response to recognize intracellular ions Hg^2+^ [93]. High-purity TPE-based peptide chemosensors with a 69% yield were generated with flexibility in solid-phase synthesis employing Fmoc(Fluorenylmethyloxycarbonyl) chemistry [93]. Following deprotection, Fmoc−L−Ser(tBu)−OH was added, and TPE, HOBt, and DIPC were coupled in DMF for four hours at room temperature. There have been schemes of TPE fluorophore synthesis (Scheme 1) [94].

**Figure 2:**
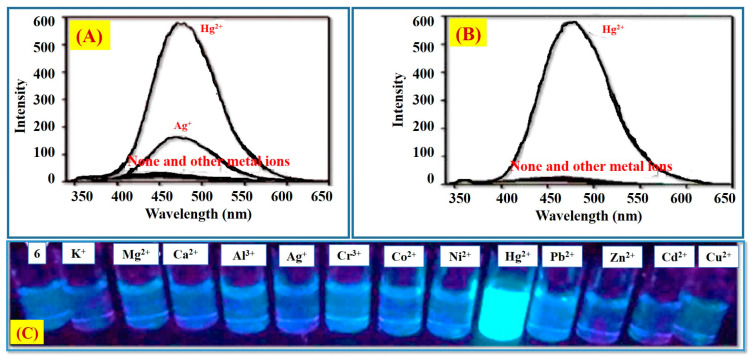
The fluorescence emission spectra of compound **6** (3 × 10^−6^ M) are shown. (**A**) Compound **91** dissolved in 10 mM phosphate at a pH of 7.4; (**B**) compound **6** was dissolved in 10 mM phosphate and 1 mM NaCl at a pH of 7.4; (**C**) compound **6** was visible under UV light (λem = 365 nm) in the presence of different metal ions. Reproduced from ref. [93] with permission from American Chemical Society, copyright 2016.

Figure 2A depicts the fluorescence response of chemical **6** to 16 metal ions. Compound **6** showed selective turn-on fluorescence responses to Hg^2+^ and Ag^+^ ions in aqueous buffered solutions after being excited at 320 nm. Figure 2B indicates that compound **6** has a unique selectivity for Hg^2+^ ions in aqueous buffered solution, differentiating it from other heavy and transition metal ions. The selective response of compound **6** to Hg^2+^ ions was visually confirmed under UV light, exhibiting a distinct light-cyan color change in the presence of Hg^2+^, whereas other metal ions showed no significant color change (Figure 2C).

**Scheme 2:**
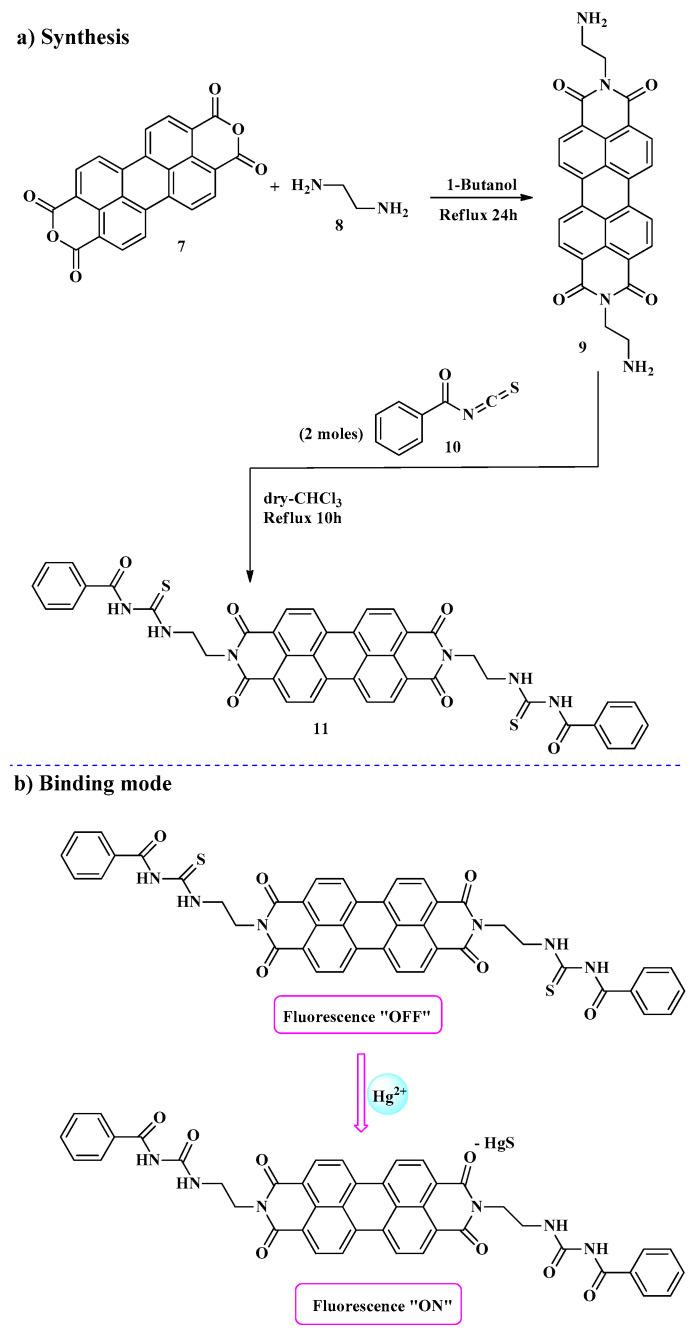
(**a**) Synthesis of sensor **11**; (**b**) Proposed binding mode.

Utilizing ^1^H NMR spectroscopy, the binding mechanism of **6** with Hg^2+^ was studied, and the binding mechanism of compound with Hg^2+^ was planned. The peptide receptor’s imidazole group was important for its interaction with Hg^2+^, and it is conceivable that the imidazole group and the peptide receptor’s C-terminal amide group cooperated to develop a stable complex. Emissions were enhanced because the 2:1 complex may agglomerate and the TPE fluorophores’ benzyl ring’s intramolecular rotation was restricted. A fluorescent chemosensor with thiourea moiety was created and manufactured recently to selectively detect mercury ions [41]. When Hg^2+^ was added, the sensor showed a color shift and a fluorescence turn-off response [95,96]. ^1^H NMR titration was used to validate the complex formation, and DFT simulations provided additional evidence. A test strip was also used for accurate and useful Hg^2+^ ion detection in semi-aqueous media [97,98]. Using a step-by-step process, a new chemosensor **11**, was created, producing an 82% yield of a highly purified dark-red solid (Scheme 2) [55].

A typical Hg^2+^-promoting desulfurization reaction, which has been studied using Job’s plot titration, HRMS, FTIR, and ^1^*H*-NMR analyses may quickly monitor Hg^2+^ in a DMSO/H_2_O (5/1, *v*/*v*) solution using a novel “turn-on” perylenebisimide-thiourea fluorescent sensor **11**. When Hg^2+^ is present, there is a notable increase in fluorescence emission at 540 and 580 nm that is highly sensitive and selective to the naked eye. Additionally, sensor **11** has a rapid response time (less than a minute) and robust anti-interference recognition. Sensor **11** is a good option for Hg^2+^ detection without a buffer system because of its quick fluorescence response and low limit of detection (0.35 μM) throughout a broad pH range of 3.0–11.0. Moreover, chemosensor **11’s** feasibility for detecting mercury ions in human liver cancer cells (HepG-2) has been investigated using fluorescent live cell imaging. It shows that the probe is not harmful to living things and that PBI-BTB has a good cell permeability for detecting mercury heavy metal ions in biological systems.

**Scheme 3:**
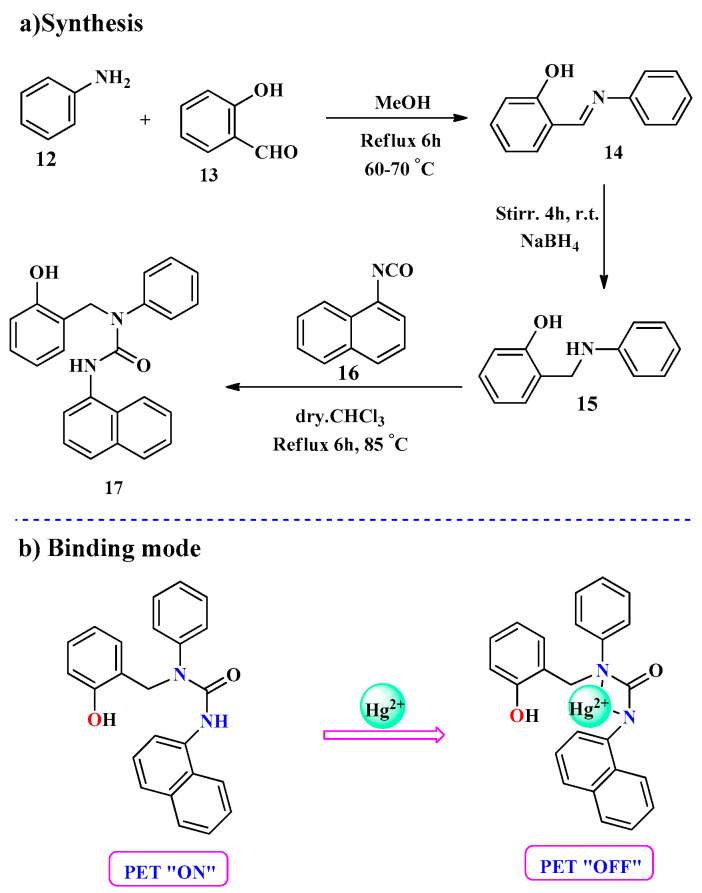
(**a**) Synthesis of thiourea-based sensor **17**; (**b**) Proposed binding mode.

J. Mishra and H. Kaur with their coworkers designed a chemosensor **17**. Salicylaldehyde and aniline were reacted in methanol, refluxed for 6 h, and then reduced with NaBH_4_ to create a Schiff base. A dark-maroon liquid was obtained by refluxing the product with naphthyl isocyanate in CHCl_3_. The finished product **17** had a 93% yield (Scheme 3) [96].

Thiourea-based chemosensor **17** demonstrated preferred recognition of Hg^2+^ in an aqueous medium, as evidenced by an increase in fluorescence emission intensity upon complexation with Hg^2+^ among numerous heavy metal ions with a detection limit of 0.84 mM. Due to the photo-induced electron transfer (PET) “OFF” mechanism at 390 nm, the suggested sensor **17** has the capacity to detect Hg^2+^ ions with excellent selectivity and sensitivity.

**Scheme 4:**
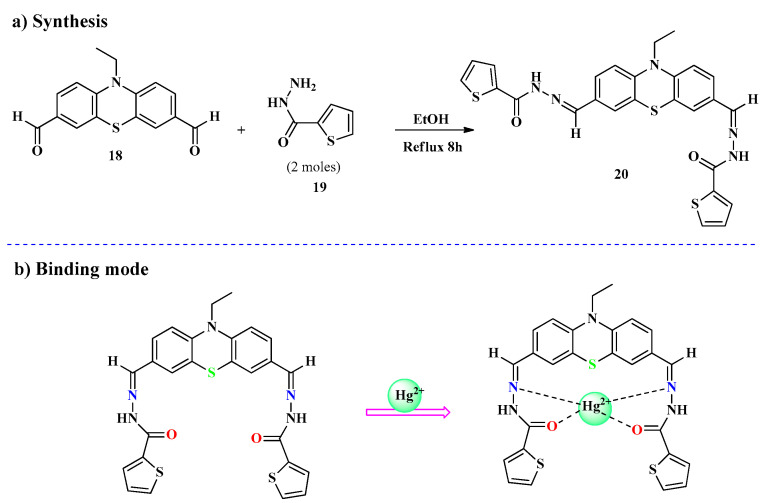
(**a**) Synthesis of phenothiazine-thiophene hydrazone-based sensor **20**; (**b**) Proposed binding mode.

V. Govindasamy et al. synthesized chemosensor **20** for detection of Hg^2+^. Thiophene-2-carboxylic acid hydrazide **19** and 10-Ethyl-10H-phenothiazine-3,7-dicarbaldehyde **18** were reacted in ethanol, refluxed for 8 h. The target product **20** with a 92% yield was obtained (Scheme 4) [99].

The synthesized sensor 20 functioned as a fluorescence “on–off” sensor to detect Hg^2+^ ions selectively in the presence of other metal ions. Furthermore, the color shift from pale green to bright yellow indicates that 20 has bound to mercury ions. The limit of detection is 0.44  ×  10^−8^ M. These findings indicate that the considered chemosensor was appropriate and capable of detecting Hg^2+^ ions from environmental water samples, with no interference from other predicted metal ions.

### 2.2. Ag^+^ Ions Detection

**Scheme 5:**
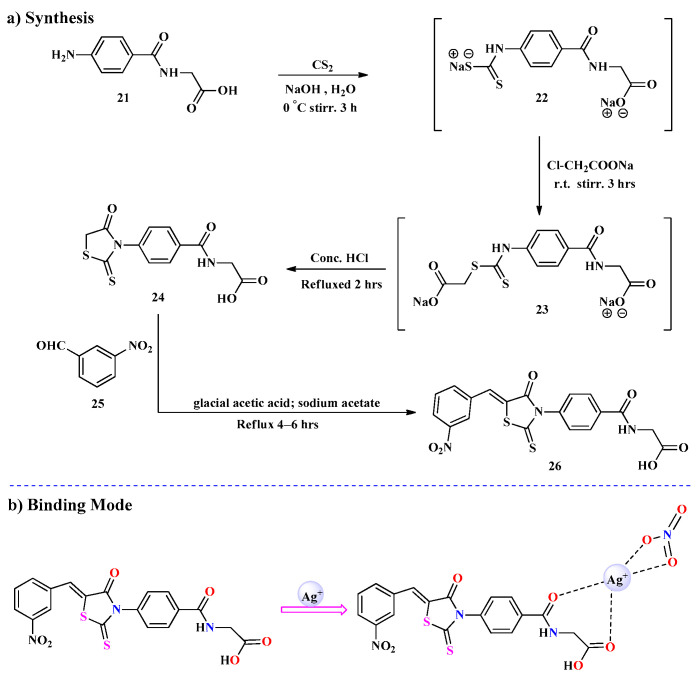
(**a**) Synthesis of rhodamine-derived fluorescent sensor **26**; (**b**) Proposed binding mode.

Silver metal ions are captivating, expensive, and physiologically active heavy metal ions. Ag^+^ ions are found in both human and animal bodies and are fundamental to many medical procedures, including antibiotics, sterilization processes, anti-infective treatment, and antibacterial activity [33,100,101]. Silver serves as a catalyst in oxidation processes as well as in electrical and photographic imaging, accessories, and silverware production [102,103]. In addition, the Ag^+^ ions make significant contributions to environmental pollution because of the growing need for silver compounds in the increased industrial and commercial sectors [104,105]. Ag^+^ ions have been released into the atmosphere as a result of these actions, threatening both humans and aquatic life [106]. Overindulging in silver over an extended time period is ideal for precipitation and adverse effects on human health [107,108]. A potentially organic chemosensor was synthesized for widely used Ag^+^ ion identification and detection [109,110]. C. I. David with his coworkers described the synthesis and design of the fluorescence-based, rhodamine-derived chemosensor receptor **26**, which was developed to detect Ag^+^ ions in DMSO-H_2_O solution mixture with excellent sensitivity and selectivity. Through the application of the chelate-enhanced fluorescence (CHEF) mechanism, the chemosensor **26** may selectively “turn on” fluorescence for Ag^+^ ions [111]. The synthesis procedure of rhodanine-3-hippuric acid involved simple steps. In a multi-step reaction with CS_2_, chloroacetic acid, and HCl, p-Amino hippuric acid **21** was changed into rhodanine-3-hippuric acid **24** as a result. The final receptor **26**, with a 59% yield after recrystallization in ethanol, was then produced by the product’s reaction with m-nitrobenzaldehyde **25** in the presence of sodium acetate and glacial acetic acid. Scheme 5 illustrates the synthesis pathway of the receptor **26** [112].

**Figure 3:**
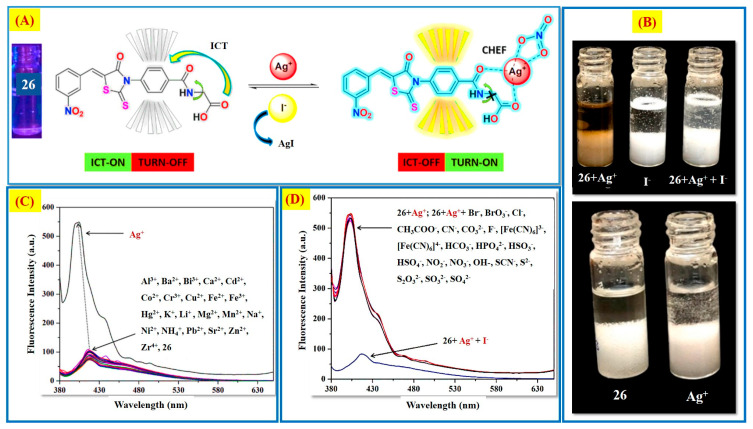
(**A**) Suggested mechanism of receptor **26** towards Ag^+^ and iodide I^−^ ions. Yellow colored arrow indiacte ICT mechanism and blue colored arrow indicate silver ion and iodide ion released as AgI (**B**) Practical naked-eye application of receptor **26** for recognition of Ag^+^ ion using solid-supported silica methods. (**C**) Fluorescence spectrum of receptor **26** (4 × 10^−6^ M). (**D**) Fluorescence spectrum of receptor **26**+Ag^+^ (4 × 10^−6^ M). The arrows indicate the fluorescence spectral lines of the respective ions. Copied from ref. [112] with permission from Elsevier, copyright 2021.

The receptor **26** exhibited a remarkable sensitivity and selectivity for the Ag^+^ ion employing a 1:1 complex stoichiometry. This was confirmed by fluorometric titrations, Job’s plot variation, Benesi–Hildebrand calculation, ^1^H-NMR, FT-IR, ICT mechanism, and DFT analyses [113]. The interaction between receptor **26** and Ag^+^ ions is distinguished by two binding phenomena: Internal Charge Transfer (ICT) and chelation-enhanced fluorescence (CHEF). Initially, receptor **26** has modest fluorescence due to free rotation of the C–N bond. Upon Ag^+^ binding, a 13 nm hypsochromic shift occurs, limiting ICT and increasing emission intensity via CHEF (Figure 3A).

The identification of Ag^+^ by receptor **26** was further verified with a solid-supported silica approach. When Ag^+^ was added to **26**-coated silica gel, the color changed from colorless to dark brown (Figure 3B). The **26**-Ag^+^ complex was completely recovered after being treated with I^−^, as evidenced by both paper strip and solid-supported silica tests [114]. The fluorescence emission of receptor **26** was measured in the presence of several physiologically and ecologically relevant metal ions in HEPES-buffered DMSO-H_2_O (pH 7.4). UV-Vis absorption measurements determined that the excitation wavelength should be 370 nm. Figure 3C shows that receptor **26** fluoresces weakly at 417 nm in the presence of other metal ions. However, the addition of Ag^+^ ions causes a 100-fold increase in fluorescence intensity at 404 nm. The **26**+Ag^+^ complex was employed to visualize anions by fluorescence emission spectroscopy. The addition of I^−^ significantly reduced the fluorescence intensity at 417 nm, but other anions had minor influence on the fluorescence intensity of **26**+Ag^+^ (Figure 3D).

**Scheme 6:**
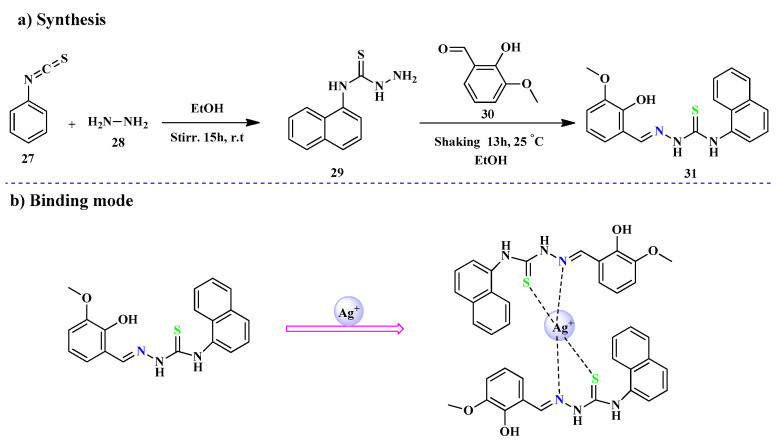
(**a**) Synthesis of naphthyl thiourea-based chemosensor **31**. (**b**) Proposed binding mode.

Y. Seo and S. Park with their collaborators designed a chemosensor **31**. 1-naphthyl isothiocyanate **27** and hydrazine **28** in ethanol interacted to form a white powder **29**. The product was reacted with O-Vanillin **30** producing intended product **31** (Scheme 6) [115]. Ag^+^ ion exhibited a selective fluorescence turn-on mechanism over the various metal ions in an efficient fluorescence naphthyl thiourea-based chemosensor **31**. It was determined that the complexation ratio of **31** with Ag^+^ was 2:1. Ag^+^ ions could be detected at low detection limits (3.82 μM) using this organic sensor (**31**).

**Scheme 7:**
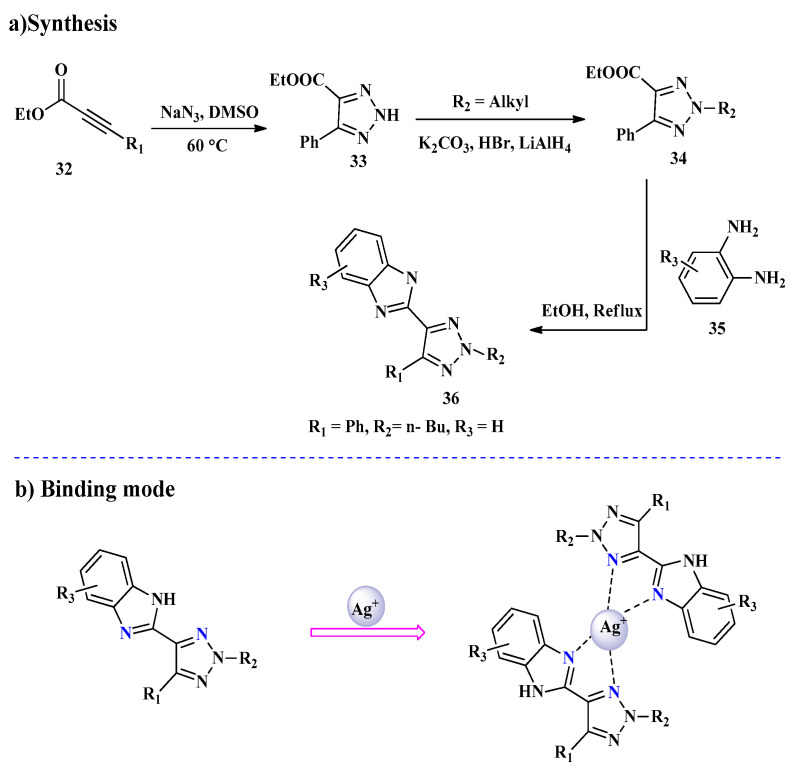
(**a**) Synthesis of triazole-imidazole-based sensor **36**. (**b**) Proposed binding mode.

Q. Lai, Q. Liu and their coworkers successfully designed and synthesized triazole-imidazole ligands 36 as selective fluorescent probes for detecting Ag^+^ ions (Scheme 7). The chemosensor 36 exhibits noticeable fluorescence emission in both aqueous and organic solutions due to the presence of intramolecular hydrogen bonding. This chemosensor is specifically useful for the evaluation of Ag^+^ ions due to its quick reaction response times in aqueous medium. The ultrafast response time (less than 30 s) of this sensor 36 in aqueous conditions demonstrated the practical application of this novel cation probe and the significance of these triazole-based fluorescent compounds in chemical and material research [116].

### 2.3. Cd^2+^ Ion Detection

**Scheme 8:**
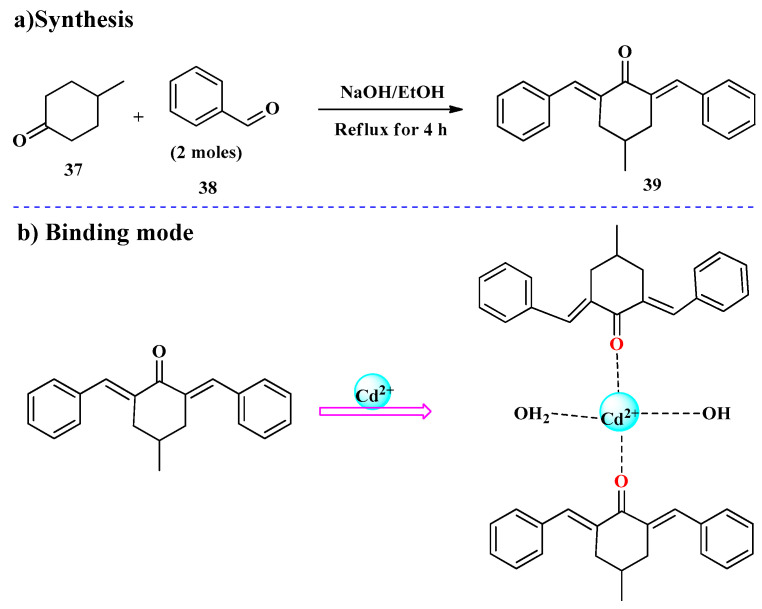
(**a**) Synthesis of optical chemosensor **39**. (**b**) Proposed binding mode.

According to the periodic table, cadmium belongs to group 12 transition metal ions. Cadmium resembles mercury and zinc metals and has a silvery-half-white appearance [117]. Cadmium is the most hazardous of the numerous toxic heavy metal ions that are found naturally in the earth’s crust [118]. Excessive cadmium exposure can cause renal failure, a drop in calcium metabolism, and the development of certain malignancies. [119,120]. One of the most hazardous and carcinogenic heavy metal ions is cadmium (Cd^2+^). Cadmium ions are used extensively in the industrial production of metal alloys, electroplating films, battery cells, and nuclear reactor control rods [121,122]. One should not undervalue the detrimental effects of Cd^2+^ ion pollution. The Cd^2+^ ion can enter the body through polluted soil, water, air, and a variety of other sources with a biological half-life of 20–30 years. This may result in several kinds of illnesses affecting the kidney, liver, lungs, heart, or other body organs [123,124,125]. The increased Cd^2+^ concentration of the body even at extremely low levels can lead to a variety of illnesses, including potentially fatal conditions such as diabetes, cancer, and chondropathy [126,127]. To obtain the best possible sensitivity and selectivity for Cd^2+^ ion detection, several photophysical characteristics must be considered while designing the construction of a fluorescent chemosensor [128]. Sadia and her colleagues developed a novel chemosensor 2, 6-di((E)-benzylidene)-4-methylcyclohexan1-one) **39**. Experimental findings demonstrated that **39** functions as an effective and selective chemosensor for Cd^2+^, even in the presence of other metal ions in the aqueous medium, comprising Hg^2+^, Cu^2+^, Mg^2+^, Ca^2+^, Al^3+^, Co^2+^, Ag^+^, Fe^3+^, Fe^2+^, Ni^2+^, Cr^3+^, Pb^2+^, Ba^2+^ and Zn^2+^. When the synthetic chemosensor **39** interacts with Cd^2+^, the fluorescence emission spectrum changes significantly. The fluorometric response verified the formation of the **39** complex with Cd^2+^. An ethanolic solution of 4-methyl cyclohexanone **37** and benzaldehyde **38** was combined with sodium hydroxide, and the mixture was refluxed for four hours at room temperature to generate the desired product **39** (Scheme 8) [129].

**Figure 4:**
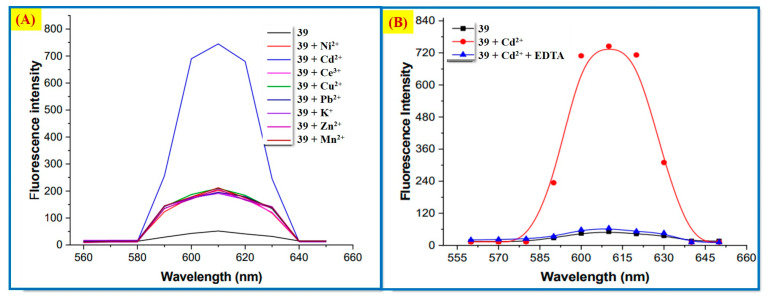
(**A**) The chemosensor **39**’s UV–visible spectrum. (**B**) The [CM1 + Cd^2+^] complex fluorescence emission spectrum. Copied from ref. [129] with permission from MDPI, copyright 2023.

The practical applications of the chemosensor **39** in the detection of Cd^2+^ ions are beneficial. To evaluate the chemosensor’s versatility, a reversibility test was performed using chelating agent EDTA in an aqueous medium at room temperature. The chemosensor can be evacuated and renewed by adding EDTA to the **39**+Cd^2+^ complex solution, as demonstrated by dropped fluorescence intensity. The addition of Cd^2+^ solution restored the enhanced fluorescence intensity. A comprehensive optical investigation was conducted to explore **39’s** specific chemosensing capabilities towards Cd^2+^. Figure 4A shows the UV-Vis absorption spectra of the **39** and **39**+Cd^2+^ complexes. The chemosensor **39** showed maximum absorbance at 390 nm due to the π-π* transition. The inclusion of Cd^2+^ increased the absorption intensity at 390 nm. The LOD of the chemosensor **39** was determined to be 19.25 Nm, as shown in Figure 4B [130].

**Scheme 9:**
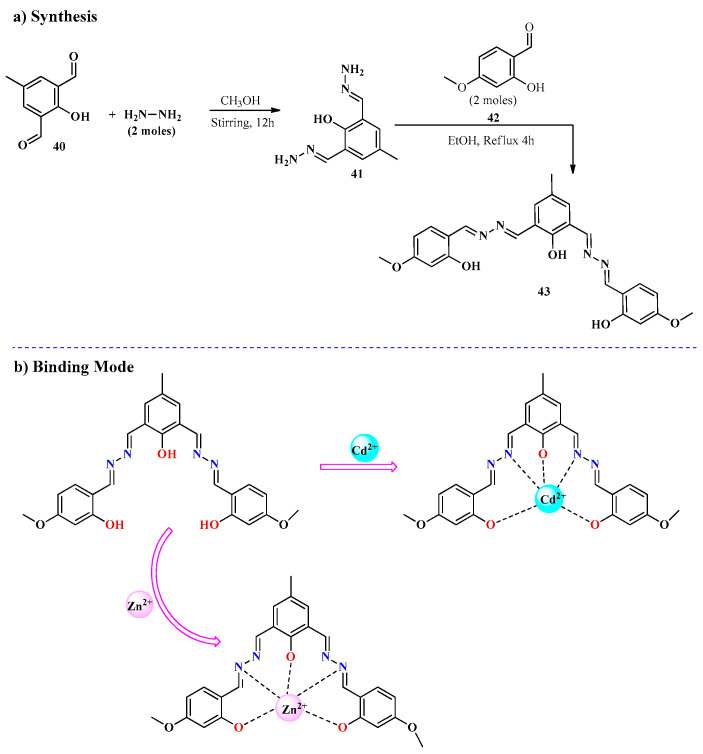
(**a**) Synthesis of Schiff-based chemosensor **43**. (**b**) Proposed binding mode.

R. Purkait, S. Dey, and C. Sinha synthesized a chemosensor **43** for detecting Cd^2+^ and Zn^2+^ ions effectively. An important aromatic aldehyde (2,6-diformylphenol) was utilized to design compartmental Schiff base ligands. Salicylaldehyde and 2,6-diformylphenol were condensed with hydrazine to produce a hetero-imine derivative (a novel Schiff base) with noticeable fluorescent properties. As 2,6-diformylphenol includes fluorophore vanillin, its vanilinyl hydrazone is a highly potent fluorogenic probe. Four nitrogen atoms and three -OH groups act as binding sites in this novel chemosensor, 6,6′-((1E,1′E)-((2E,2’E)-((2-hydroxy-5-methyl-1,3 phenylene)bis(methanylylidene))bis(hydrazine-2,1-diylidene))bis(3-methoxyphenol). The synthesis procedure involved reacting 4-methyl-2,6-diformylphenol **40** with excess hydrazine in MeOH, and then condensing the resulting 2,6-bis((E)-hydrazonomethyl)-4-methylphenol **41** with 2-hydroxy-4-methoxybenzaldehyde **42** in dry EtOH. This produced 88% yield of the intended product **43** (Scheme 9) [119].

A highly effective fluorescent sensor 43 for the detection of Cd^2+^ in DMSO-aqueous (9:1, *v*/*v*) HEPES buffer (pH, 7.2) is demonstrated using the visible-light-excitable sensor. Additionally, a recovery study from drinking water and a test kit employing a TLC plate were carried out effectively to demonstrate its usefulness in real-world applications.

### 2.4. Pb^2+^ Ion Detection

**Scheme 10:**
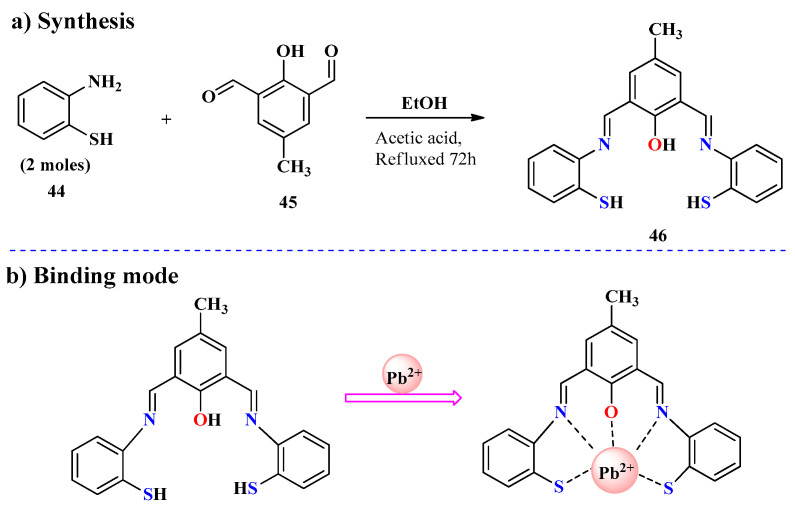
(**a**) Synthesis of Schiff-based chemosensor **46**. (**b**) Proposed binding mode.

Lead (Pb) is a common heavy metal that finds use in the current industrialized world including paints, fuel, batteries, and other materials [131,132,133]. In several domains, Pb^2+^ is essential as a transitional element [134]. The production of lead nitrate for commercial use began in the 19th century, according to lead history [135]. At the time, it was mostly utilized in the industrial sector as the primary raw material for pigment production [136]. Lead (Pb) made a substantial contribution to industrial output. In the industrial sector, it was used as a heat stabilizer in thermal imaging paper, nylon, and polyester coatings [137,138,139]. According to recent research, lead (Pb) and its compounds enter the body through wastewater and waste residues and harm the kidney, brain, digestive system, and other organs and systems [140]. One of the most damaging heavy metal pollutants in the environment is the lead ion (Pb^2+^), which constitutes significant risks to all living things and generates persistent problems, particularly with regard to human health and animal [141,142]. Even at extremely low levels, chronic exposure causes a variety of health issues, including neurological illnesses, cardiovascular diseases, fertility diseases, and hypertension [143,144]. Keeping in view the above-mentioned health threat associated with lead poisoning, it is of key relevance to detect and determine its presence in various environmental and biological samples [145,146,147,148]. Pb^2+^ ions could possibly be found and determined using a variety of methodologies, including the most potent, precise, and practical colorimetric and fluorescent sensing technologies [149,150]. 2-Aminothiophenol 44 and 2-hydroxy-5-methylbenzene-1,3-dialdehyde 45 were added in ethanol and a catalytic amount of acetic acid. The resultant mixture was refluxed for 72 h. The precipitate of 46 was then collected on filter paper (Scheme 10) [151].

**Figure 5:**
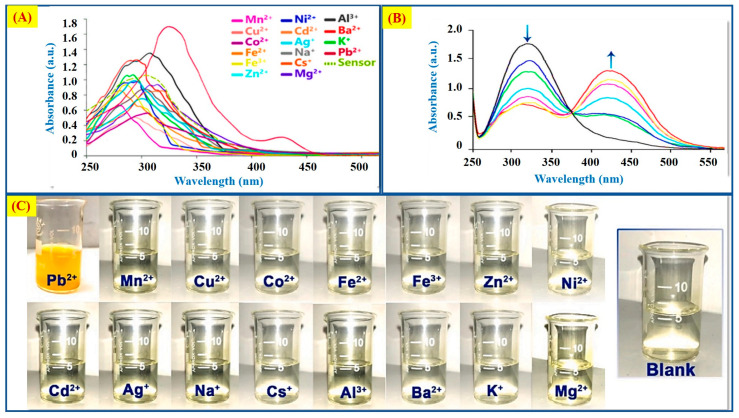
(**A**) The UV–Vis absorption spectrum of the chemosensor **46** in the presence of different cations. (**B**) The UV–Vis spectrum of **46** titration against various concentrations of Pb^2+^. The arrow indicate the UV-Vis spectrum of the chemosensor 46 titration against various concentrations of lead ion. (**C**) Sensor **46** color change in the presence of various cations in an ethanol–water solution (*v*/*v*, 90, 10). Copied from ref. [151] with permission from Elsevier, copyright 2023.

Figure 5A shows the UV-Vis absorption spectra of chemosensor **46**, with a maximum absorption wavelength (λ_Abs max_) of 300 nm. The addition of Pb^2+^ ions changed the λ_Abs max_ to 325 nm and developed a new absorption band at 430 nm. In contrast, other ions had no appreciable effect on the absorption spectrum of **46**. Sensor **46** solutions (4 × 10^−5^ M) in ethanol were titrated with Pb^2+^ ions (0–6 equivalents). As Pb^2+^ concentration increased, absorption strength at 325 nm dropped, but a significant charge-transfer band appeared at 430 nm (Figure 5B). Sensor **46** (15 μM) underwent colorimetric experiments with different metal ions. After adding 4 equivalents of Pb^2+^ ions, the colorless solution became purple; Figure 5C indicates significant Pb^2+^ selectivity. The color shift is due to ligand-to-metal charge transfer (LMCT).

A unique fluorescent chemosensor **49** for the detection of Pb^2+^ ion was successfully designed. Compound **47** was reacted with N-hydroxysuccinimide and DCC in dichloromethane. Triethylamine and compound **48** were then added. The liquid was stirred overnight. DCM/EtOH was used as the eluent in column chromatography to purify the crude product 49 [152].

**Scheme 11:**
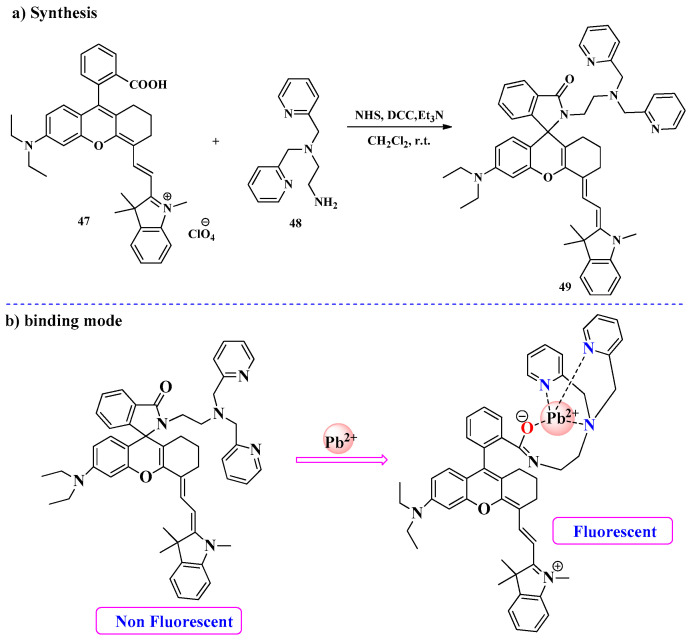
(**a**) Synthesis of near-infrared fluorescent probe **49**. (**b**) Proposed binding mode.

Sensor **49** demonstrated exceptional selectivity for Pb^2+^ ions over a wide range of other metals (Scheme 11). Sensor **49** turns from colorless to green when Pb^2+^ is added, gaining absorption in the near-IR portion of the spectrum. In acetonitrile, it also exhibits intense “off–on” fluorescence along with a color shift from colorless to fluorescent pink. The reversible spirocyclic ring-opening mechanism upon binding of Pb^2+^ ions was responsible for the observed spectrum changes. The concentration of Pb^2+^ ions is proportional to the lead-induced absorbance at 718 nm and the fluorescence intensity at 735 nm. This suggests that the lead ions in the solution or living cells can be quantified using colorimetric or fluorometric methods. Thus, **49** may be used as a potential lead chemosensor in medical research and environmental monitoring.

**Scheme 12:**
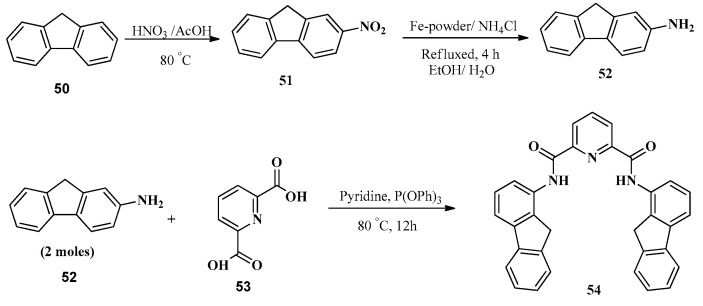
Synthesis of pyridine-2,6-dicarboxamide-based chemosensor **54**.

The cation receptor **54** was designed with -nitro-9H-fluorene **51**. After that, iron powder and ammonium chloride were used to convert it to 2-amino-9H-fluorene **52**. Lastly, the desired product **54** with a 74% yield was obtained by coupling with pyridine-2,6-dicarboxylic acid **53** in the presence of triphenyl phosphite (Scheme 12) [142]. The metal ion binding ability of the chemosensor 54 was studied in the presence of various metal ions using UV-vis and fluorescence studies, and the results showed that the chemosensor 54 has a suitable selectivity and considerable sensitivity for the detection of Pb^2+^ ions. The 54+Pb^2+^ complexes were found to have an association constant (K_a_) of 5.65 × 10^8^ M^−2^. The significant sensitivity of the chemosensor 54 was evident from the acquired limit of detection (LOD) values (2.31 × 10^−6^ M for Pb^2+^).

### 2.5. In^3+^ Ion Detection

**Scheme 13:**
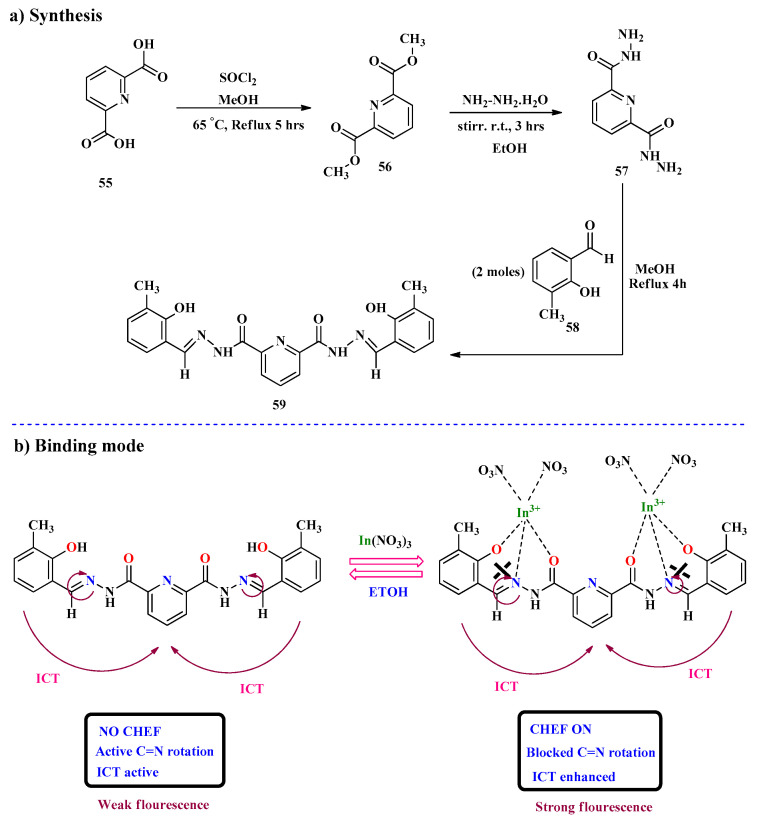
(**a**) Synthesis of pyridinecarbohydrazide-based fluorescent chemosensor **59**. (**b**) Proposed binding mode.

The Group 13 heavy metal in the periodic table, indium (In), is extensively used in many applications, particularly in the industrial sectors [153], novel semiconductors [154], solar cell batteries [155,156], and gas sensors [157]. Apart from these uses, its pollution might have serious adverse consequences for health [158]. It is known to disrupt the metabolism of ferric iron in cells at the locations of usage, storage, and transport. It has been shown to have negative effects on humans even though it has no biological purpose in the human body [159]. Additionally, studies on animals have demonstrated that acute intravenous injections of indium compounds pose a serious risk to the liver and kidney [160]. Numerous analytical techniques, such as electrothermal atomic absorption spectroscopy [161], adsorptive stripping voltammetry, spectrophotometry [162], and inductively coupled plasma mass spectrometry [163], have been employed to detect indium [164]. However, the methods involve laborious, time-consuming procedures and are comparatively expensive [165]. These factors make the development of chemosensors that can detect indium essential [166,167]. However, compared to other metal ions, chemosensors for In^3+^ are rare [168]. Furthermore, it is difficult to differentiate In^3+^ from Al^3+^ because of their similar chemical properties [164]. On the other hand, chemosensors have a number of benefits, such as affordability, usability, and quick reaction. The most effective option to recognize In^3+^ ions in living things is utilizing fluorescent chemosensors [169]. In order to detect and quantify In^3+^ ions, Aatif et al. synthesized and characterized an innovative probe, N,N-bis((E)-2-hydroxy-3-methyl benzylidene)pyridine2,6-dicarbohydrazide **59**. When the In^3+^ ion is introduced to DMSO: H_2_O medium, probe **59** exhibits a powerful ON fluorescence with a low detection limit. To obtain the desired end product **59**, a methanolic solution of 2-hydroxy-3-methylbenzaldehyde **58** was treated with pyridine-2,6-dicarbohydrazide **57**. The entire mixture was refluxed for five hours. A white powder with an 88% yield was obtained by filtering the product and then washing it with an excessive amount of methanol (Scheme 13) [161].

**Figure 6:**
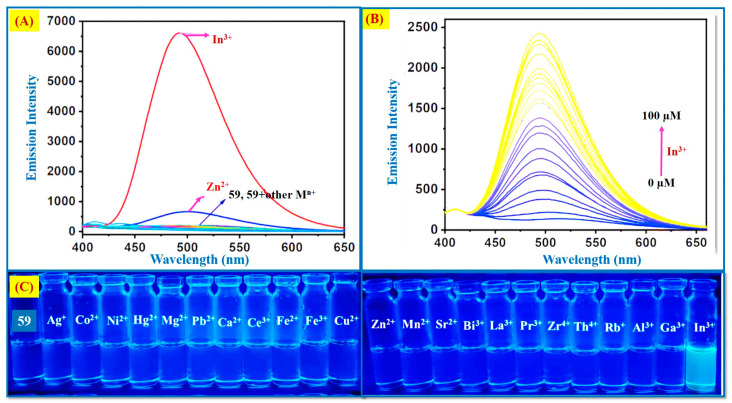
(**A**) Fluorescence spectrum responses of **59** in DMSO: H_2_O when it is excited using different metal ions at 337 nm. (**B**) Probe **59’s** fluorescence spectrum titration in DMSO:H_2_O (2:8, *v*/*v*) medium. (**C**) Under UV light, the fluorescence of **59** in a DMSO:H_2_O solution containing different metal ions changes. Copied from ref. [161] with permission from Elsevier, copyright 2022.

Probe **59** demonstrates preferential binding with In^3+^, and the complex reveals strong fluorescence intensity. This could possibly be the result of the simultaneous involvement of crucial aspects that need to be taken into account. For example, a process known as intramolecular charge transfer (ICT) may be responsible for the sharp rise in emission intensity, independent of red shift [170]. Probe **59** is dimly fluorescent because it has > C=N–, which promotes > C=N rotation [171]. Rotation is prohibited, and fluorescence characteristics are boosted upon interaction with In^3+^ ions. The coordination complex forms an extremely rigid structure that hinders nonradioactive decay. It is the source of the chelation-enhanced fluorescence effect (CHEF) [172]. Figure 6A illustrates that, after excitation at 337 nm, the emission peak **59** occurs at 500 nm. Fluorescence abruptly increased by 60 times when In^3+^ (10 eq.) was added. Furthermore, when Al^3+^ and Ga^3+^ were analyzed with the other tested ions, no appreciable variation in emission intensity was observed. These results suggest that In^3+^ ions may be detected using colorimetric and fluorescence characteristics. A spectral titration was used to investigate the linear connection between concentrations and intensity in order to assess probe sensitivity (Figure 6B). The spectral titration of **59** (50 μM) with In^3+^ was carried out by gradually adding 0–60 μM in DMSO: H_2_O (2:8, *v*/*v*) medium. When In^3+^ is added to a solution containing **59**, a new emission band appears at about 500 nm, and the fluorescence intensity changes when excited at 337 nm. The performance of L (50 μM) in DMSO: H_2_O (2:8, *v*/*v*) is shown, when reacted with 10 equivalents of different metal ions such as Ag^+^, Rb^+^, Ca^2+^, Mg^2+^, Co^2+^, Ni^2+^, Mn^2+^, Hg^2+^, Pb^2+^, Cd^2+^, Zn^2+^, Cu^2+^, Ba^2+^, Sr^2+^, Fe^3+^, Al^3+^, Ga^3+^, In^3+^, La^3+^, Ce^3+^, Pr^3+^, Bi^3+^, Zr^4+^, and Th^4+^, (Figure 6C). Under normal conditions, the color of the solution in any glass vial does not change; nevertheless, when exposed to UV light, only the vial containing **59** with In^3+^ ions show luminous activity, whilst the others do not. This shows that **59** is selective for In^3+^ ions. Furthermore, its absorption and emission spectrum responses were thoroughly investigated. Sensor **59** may be used to detect In^3+^ ions in environmental samples, as evidenced by the detection of In^3+^ in water samples. Interestingly, the bio-imaging showed that 59 was able to identify In^3+^ ions in zebrafish larvae and DrG cells.

### 2.6. Simultaneous Multiple-Ion Detection

**Scheme 14:**
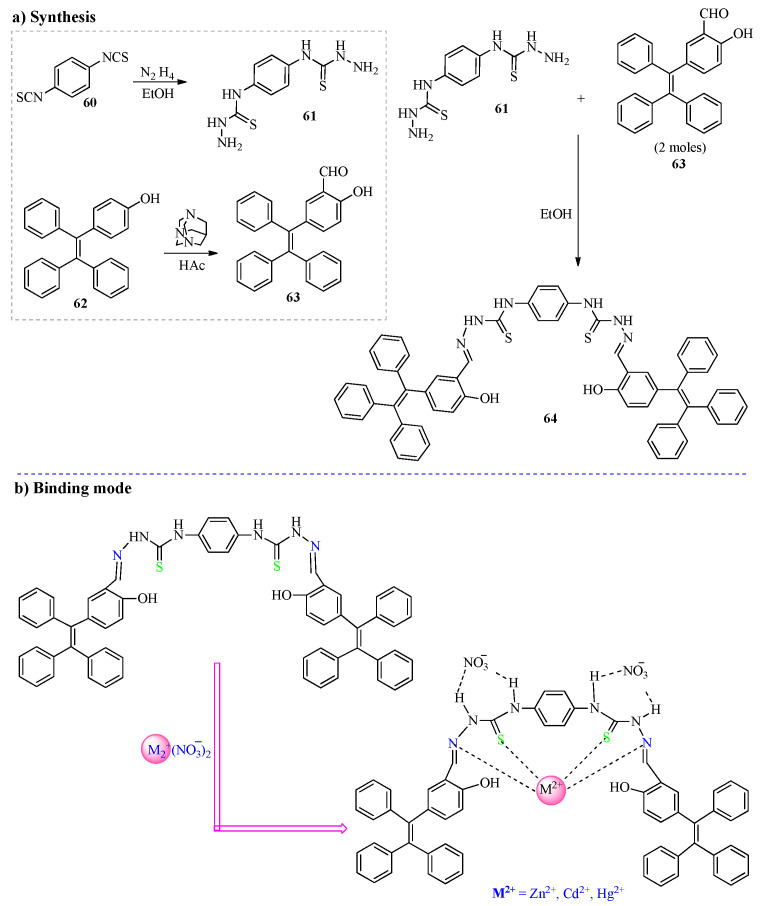
(**a**) Synthesis of AIE-based fluorescence sensor **64**. (**b**) Proposed binding mode.

Several heavy metal ions in the environments and living bodies are detected by the synthesis of a basic and highly efficient fluorescence chemosensor that can sense Zn^2+^, Hg^2+^, and Cd^2+^ simultaneously [117]. It is commonly known that aggregation-caused quenching (ACQ) is the phenomenon that leads fluorescence emission of organic fluorophores to frequently get quenched in the aggregated form [173]. The ACQ effect has significantly limited the usage of several organic fluorophores in organic light-emitting diodes and as sensing materials (chemosensors, biosensors) [174]. To lessen the ACQ effect, dendritic wedges, branching chains, and massive cyclic species have been covalently linked to the fluorophores to prevent aggregation formation [175]. The first AIE-based fluorescence sensor **64** that may recognize Zn^2+^, Hg^2+^, and Cd^2+^ heavy metal ions in aqueous solutions at the same time is presented in this strategy [176,177]. Formyltetraphenylethylene and the bis-thiosemicarbazide derivative underwent a condensation process to create thiourea-bridging bis-tetraphenylethylene (Bis-TPE) with 82% yield (Scheme 14). In aqueous medium, at 500–526 nm, the long-wavelength fluorescence was evident and was generated by the AIE effect. They showed high selectivity for Hg^2+^ sensing with fluorescence amplification, high selectivity for Cd^2+^ sensing with red to yellow fluorescence change, and high selectivity for Zn^2+^ sensing with red to green fluorescence change. Furthermore, with the help of Cl^−^ and ATP, it was able to concurrently detect Zn^2+^, Cd^2+^, and Hg^2+^ in the combined solution of these three types of metal ions by altering the fluorescence [178].

**Figure 7:**
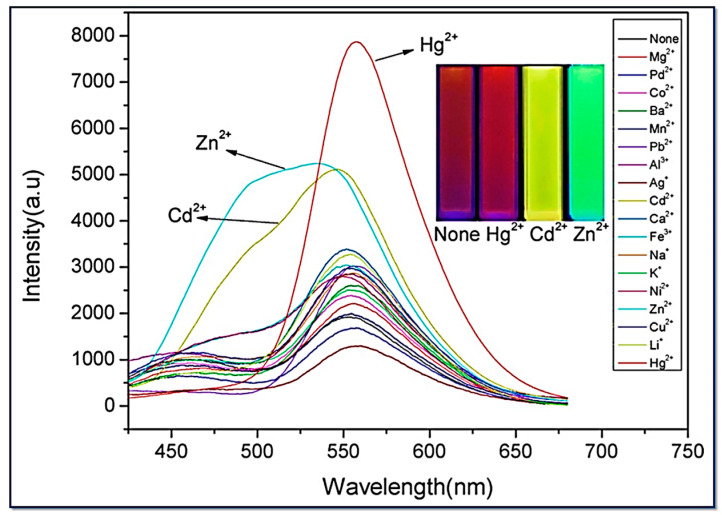
Bis-TPE’s (**64**) emission intensities with various metal ions in an H_2_O-THF solution. Copied from ref. [178] with permission from Elsevier, copyright 2020.

As H_2_O-THF solution (which contains 90% H_2_O) demonstrated the highest fluorescence emission, this solution system was selected as the aqueous medium to examine chemosensor **64’s** sensing capacity for various metal ions. Figure 7 displayed the sensing fluorescence spectra. The addition of the metal ions Mg^2+^, Pd^2+^, Co^2+^, Ba^2+^, Mn^2+^, Pb^2+^, Al^3+^, Ag^+^, Ca^2+^, Fe^3+^, Na^+^, K^+^, Ni^2+^, Cu^2+^, and Li^+^ was seen to cause no discernible change in the fluorescence wavelength and a narrow range of emission intensities. However, a notable alteration in the fluorescence emission was noted upon the addition of Zn^2+^, Cd^2+^, or Hg^2+^. The fluorescence rose significantly with the addition of Hg^2+^, while the maximum emission wavelength remained at around 550–580 nm. When Zn^2+^ was added, the fluorescence emission significantly increased and the highest emission wavelength shifted from 550–580 nm to 500–550 nm. Additionally, the solution’s color changed from red to green. The fluorescence showed a shift from 550–580 nm to 500–580 nm with a noticeable increase in emission strength and a color change from red to yellow upon the addition of Cd^2+^ Bis-TPE **64** showing strong selective sensitivity to Zn^2+^, Cd^2+^, and Hg^2+^, respectively, as demonstrated by these data.

**Scheme 15:**
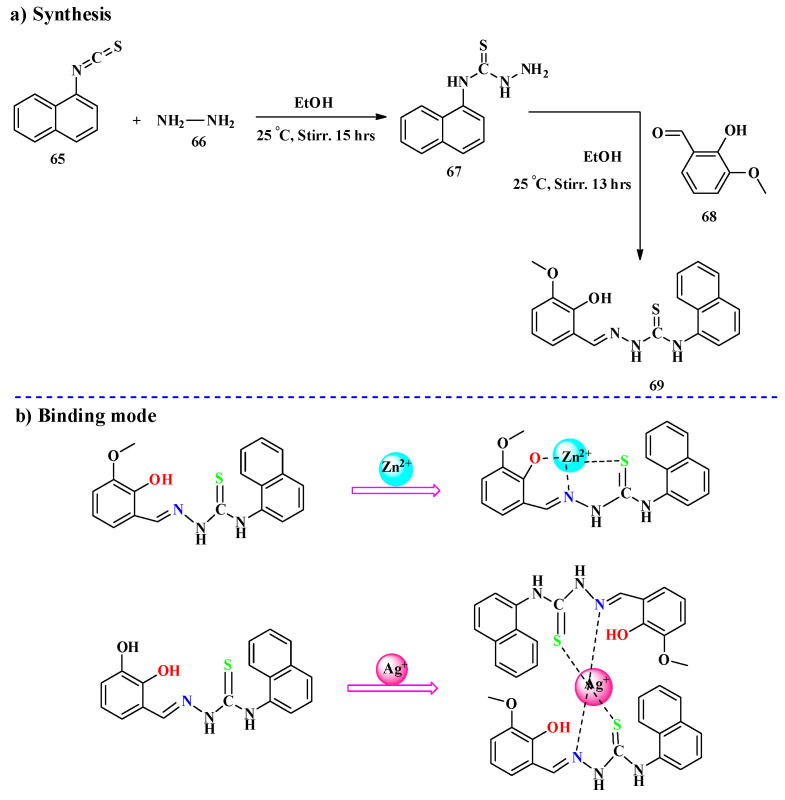
(**a**) Synthesis of naphthyl thiourea-based chemosensor **69**. (**b**) Proposed binding mode.

Y. Seo, S. Park, and their colleagues reported the synthesis of a naphthyl thiourea-based chemosensor **69** that demonstrates a powerful fluorescence turn-on response to detect Ag^+^ and Zn^2+^ ions in specific solvent conditions. They initiated the reaction by mixing the hydrazine **66** and 1-naphthyl isothiocyanate **65** in ethanol. The required product with a 30% yield was obtained by reacting the white powder with O-vanillin **68** in methanol (Scheme 15) [115]. Chemosensor **69** demonstrated rapid reactions to Ag^+^ and Zn^2+^ by turning on a noticeable fluorescence under various solvent conditions. It was discovered that the binding ratio of **69** to Ag^+^ and Zn^2+^ were 2:1 and 1:1, respectively. The HNC detection limits for Ag^+^ and Zn^2+^ were determined to be 3.82 and 0.21 μM, respectively. The binding mechanisms of **69** for Ag^+^ and Zn^2+^ were illustrated using Job’s plot.

**Scheme 16:**
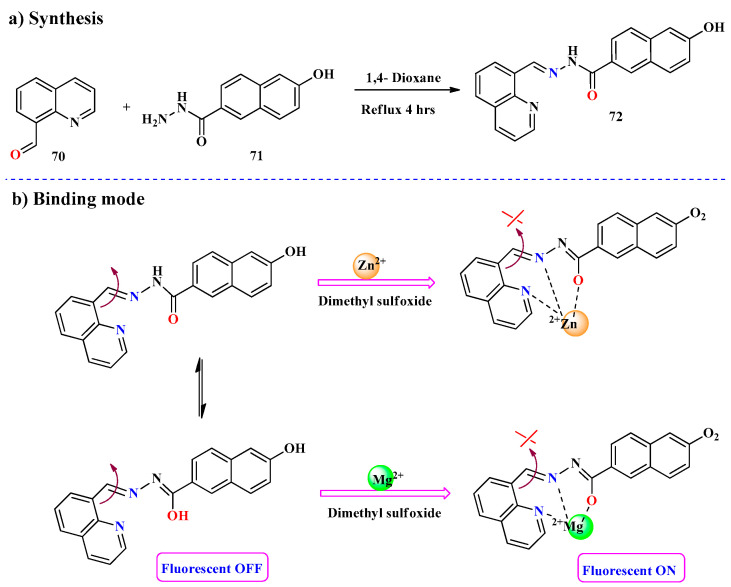
(**a**) Synthesis of Schiff-based chemosensor **72**. (**b**) Proposed binding mode.

Y. Ding, C. Zhao, and their collaborators developed an efficient detection chemosensor for Zn and Mg metal ions. A chemosensor 6-hydroxy-N′-(quinolin-8-ylmethylene)naphthalene-2-carbohydrazide **72** was synthesized by refluxing quinoline-8-carboxaldehyde **70** and 6-hydroxy-2-naphthoic hydrazide **71** in 1,4-dioxane for 4 h, yielding 78% of the intended product **72** (Scheme 16) [179]. Sensor **72** demonstrated quick identification of Zn^2+^ in DMSO/H_2_O (4:1, *v*/*v*) and Mg^2+^ in ethanol/water (9:1, *v*/*v*) as dual purpose turn-on fluorescence chemosensor. The coordination reaction between **72** and the target ions, which encouraged intramolecular charge transfer and inhibited the C=N isomerization process, was linked to the improvement in fluorescence detection. At the same time, a quick color shift from colorless to yellow or yellowish-green under UV light (365 nm) was readily apparent to the naked eye. The limits of detection and quantification under ideal circumstances were 32.3 nM and 97.8 nM for Zn^2+^ and 16.1 nM and 48.9 nM for Mg^2+^, respectively.

**Scheme 17:**
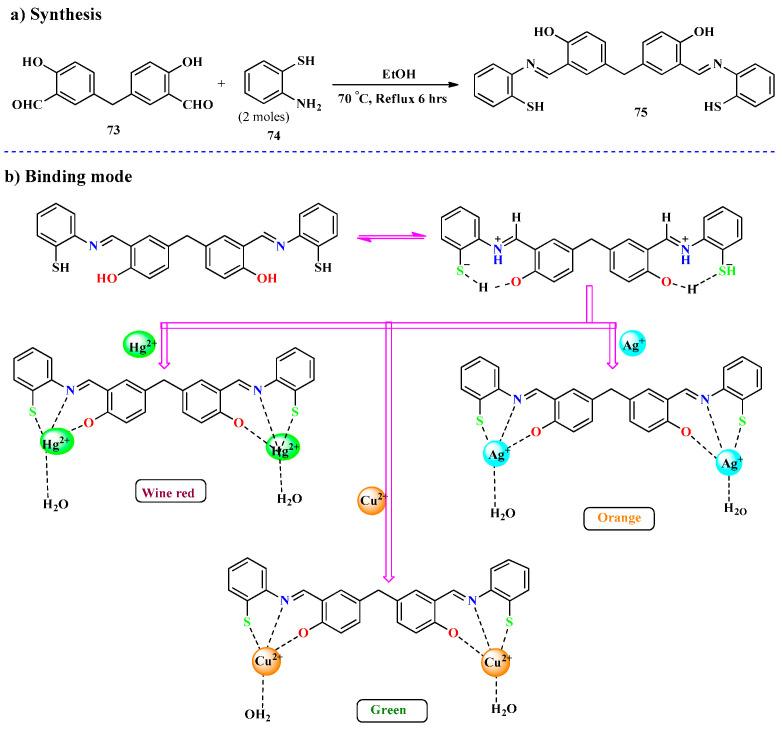
(**a**) Synthesis of Schiff-based chemosensor **75**. (**b**) Proposed binding mode.

S. Zhang, X. Wu, and their coworkers reported a novel, reversal chemosensor based on a simple Schiff base that demonstrates both colorimetric and fluorescent characteristics. The chemosensor exhibited great selectivity and sensitivity towards Ag^+^, Cu^2+^, and Hg^2+^ metal ions in a DMSO/H_2_O solution, and its quick response time permits for visual identification or detection by the naked eye. The synthesis of the sensor 4,4′-methylenebis [2-[[(2-mercaptophenyl)imino]methyl]phenol **75** includes refluxing the solution of 5,5′-methylenebis(2-hydroxybenzaldehyde) **73** and 2-aminobenzenethiol **74** in EtOH for six hours (Scheme 17). The resultant product was cooled, filtered, and recrystallized from DMF (dimethyl formamide), yielding the intended product **75** with an 85% yield [180]. Sensor **75** showed good selectivity and sensitivity to Ag^+^, Cu^2+^, and Hg^2+^ ions in DMSO/H_2_O (1/1, *v*/*v*) solution among diverse metal ions, with results that could be observed immediately with naked eyes. According to Job’s plot and FT-IR analysis, the binding stoichiometry between Ag^+^/Hg^2+^/Cu^2+^ and **75** is 1:2. Ag^+^, Cu^2+^, and Hg^2+^ could be detected by MMIP throughout a broad pH range of 3–10. For Ag^+^, Cu^2+^, and Hg^2+^, excellent linear relationships were found in concentration ranges of 0–20 μM. Therefore, **75** could be used successfully to measure the levels of Ag^+^, Cu^2+^, and Hg^2+^ in aqueous solution.

## 3. Limitations and Challenges of the Current Chemosensors

Chromofluorescent chemosensors are essential for offering quick, affordable, and easy solutions to challenging issues because of their versatility, high sensitivity, and selectivity [181,182]. New frontiers in the discipline have also been made possible by the development of sophisticated data analysis methods, better apparatus, and innovative sensor materials [183]. Efforts must be made to develop and implement fluorescence probes that can function in pure aqueous solutions with physiological pH. However, certain chemosensors have several limitations. For example, synthesis of a stable and highly selective chemosensors can be difficult and expensive. Certain chemosensors express changes in fluorescence due to temperature, pH or ionic strength variations rather than specific analyte interactions. The fluorescence response may become saturated at high analyte concentrations, rendering them unsuitable for detecting a wide range of concentrations. The low water solubility of many organic-dye-based sensors restricts their use in biological systems. The potential for cytotoxicity in certain sensor materials restricts their usage in biological applications. A common problem with chromofluorogenic chemosensors is cross-reactivity, which causes them to react to several analytes, hence decreasing specificity. A large number of organic chemosensors are irreversible, which means that repeated or continuous measurements are not appropriate for them. The long-term use of certain fluorophores is limited by photobleaching. Others may disintegrate in harsh chemical environments. Some sensors detect target analytes with slow kinetics, making them suitable for real-time monitoring applications. Although these are extremely sensitive in controlled circumstances, other molecules’ interference can cause them to perform inefficiently in biological fluids or environmental samples. Researchers are actively attempting to address these restrictions through molecular engineering, nanotechnology, and hybrid material techniques.

## 4. Conclusions and Future Perspectives

In this study, different synthetic approaches of chromofluorescent chemosensors based on organic compounds, including Schiff base, naphthyl thiourea, perylenebisimide-thiourea, thiourea, phenothiazine-thiophene hydrazone, rhodamine-derived, triazole-imidazole, pyridine-2,6-dicarboxamide, pyridinecarbohydrazide, solid-phase peptide, optical, near-infrared, AIE-based and other functional groups, have been reviewed for the detection of selected heavy metal ions. Chromofluorescent sensing technology is a highly active field of study. We classified colorimetric and fluorometric sensors for the detection of heavy metal ions based on their receptors into several categories, such as Hg^2+^, Ag^2+^, Cd^2+^, Pb^2+^, In^3+^, and simultaneous multiple-ion detection. The development of fluorescent chemosensors is clearly far from completion [184]. Their capabilities could be further improved by emerging technologies like fluorescence-based sensors. Furthermore, point-of-care diagnostics and customized healthcare are being revolutionized by the incorporation of these sensors into portable devices [181,185]. The current review article examined new developments in the field of environmental monitoring for heavy and transition metal ions as well as possible future directions. This has increased the review’s scope and significance for readers who are curious about the broader background of environmental monitoring. The development of such sensors would aid in developing quick and simple solutions to a number of problems relating to the identification of toxic metal ions at trace levels in a range of biological materials. In order to detect and identify heavy metal ions, we hope that this article can assist readers in the future with the development of highly sensitive, selective, and effective chemosensors. The purpose of this study is to stimulate further research and advancement in the fields of analytical chemistry and other fields by providing inventive solutions to a variety of real-world problems.
